# Effect of methimazole treatment on Th1, Th17, and Th22 lymphocytes in pediatric Graves’ disease patients

**DOI:** 10.3389/fimmu.2024.1431686

**Published:** 2024-10-03

**Authors:** Aleksandra Starosz, Karolina Stożek, Aleksandra Opęchowska, Filip Bossowski, Marcin Moniuszko, Kamil Grubczak, Artur Bossowski

**Affiliations:** ^1^ Department of Regenerative Medicine and Immune Regulation, Medical University of Bialystok, Bialystok, Poland; ^2^ Clinical Department of Pediatrics, Endocrinology, Diabetes with Cardiology Division, Medical University of Bialystok, Bialystok, Poland; ^3^ Department of Allergology and Internal Medicine, Medical University of Bialystok, Bialystok, Poland

**Keywords:** Graves’ disease, methimazole treatment, Th1 lymphocytes, Th17 lymphocytes, Th22 lymphocytes

## Abstract

Graves’ disease is the leading cause of autoimmune hyperthyroidism. Thyroid hormones are an essential element of the endocrine system, playing a pivotal role in the body’s development, especially important in children with intensified growth. Disturbance within thyroid tissue certainly affected the whole body. Nowadays, numerous research studies indicate different factors contributing to the onset of the disease; however, the exact pathomechanism of Graves’ disease is still not fully understood, especially in the context of immune-related processes. Th1, Th17, and Th22 effector lymphocytes were found to be crucial participants in the disease outcome, as well as in autoimmune diseases. Here, our study aimed at assessing selected effector T lymphocytes, Th1, Th17, and Th22, in newly diagnosed pediatric Graves’ disease patients, together with their association with thyroid-related parameters and the potential outcome of disease management. We indicated significant increases in the frequencies and absolute numbers of selected effector lymphocytes in Graves’ disease patients. In addition, their mutual ratios, as well as Th1/Th17, Th/Th22, and Th17/Th22, seem to be significant in those diseases. Notably, low Th17/Th22 ratio values were distinguished as potential prognostic factors for normalizing TSH levels in response to methimazole treatment. To sum up, our research determines the crucial contribution of Th1, Th17, and Th22 cells in the pathogenesis of Graves’ disease. Moreover, the mentioned subset of T cells is highly likely to play a substantial role in the potential prediction of therapy outcomes.

## Graphical Abstract

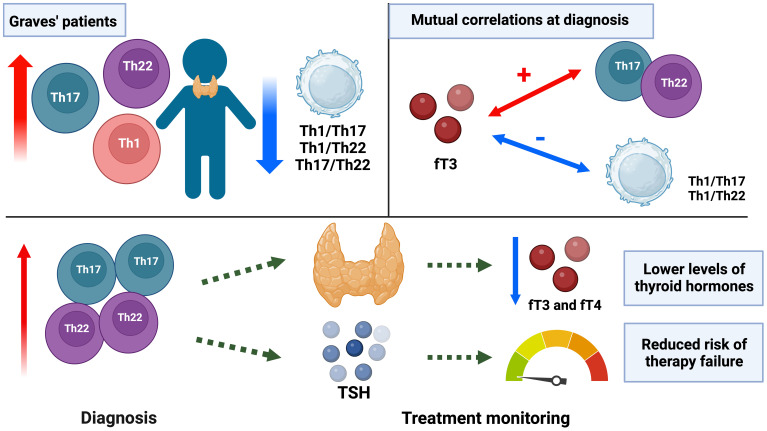

## Introduction

Graves’ disease (GD), an autoimmune thyroid disease (AITD), is the leading cause of hyperthyroidism in the adult population (1). Pediatric GD constitutes approximately 5% of all GD cases, with prevalence gradually increasing with age (2). Disturbance within thyroid tissue is associated with autoimmune processes; however, its clinical manifestations can affect the whole body. Dominant symptoms of GD are associated with the systemic excess of thyroid hormones, including *inter alia*: tachycardia, goiter, neck swelling, and exophthalmos (3). First-line therapy is based on the administration of methimazole/thiamazole to counteract the increased release of thyroid hormones, with doses strictly dependent on the disease’s advancement and severity (2, 4, 5). The pathogenesis of the disease results from autoimmune reactions, with genetic, epigenetic, and environmental factors affecting GD outcome and development. Mutation of TSH receptor gene (*TSHR*), leading to incorrectly built protein receptors and concomitant autoantibody production, was one of the first gene-related autoimmune backgrounds of GD (6). Recent studies indicated the crucial role of five alleles that are more frequent in patients with GD, class I human leukocyte antigen (HLA I B8 and Cw7) and class II (HLA II DQ2, DR3, and DR4), affecting *inter alia* elevated fT3 concentrations (7, 8). Despite the discovery of numerous factors contributing to the onset of autoimmune response, the knowledge on the sophisticated net of immune-mediated processes is still scarce.

Lymphocytes expressing CD4 (helper T cells) are significant orchestrators of immune reactions, modulating other cells’ activity through a wide range of released cytokines. In reference to autoimmunity, Th1 lymphocytes, differentiated via stimulation with IFN-gamma and IL-12, can activate macrophages and cytotoxic lymphocytes damaging the thyroid tissue. Moreover, the secretion of Th1-related cytokines like IFN-gamma and TNF-alpha can inhibit the growth of thyrocytes and reduce the binding of iodine to thyroglobulin (9, 10). Previous research demonstrated elevated values of Th1 cytokines in the serum of thyroid-associated active orbitopathy patients (11). In addition, the lack of helper T cells’ balance, especially Th1/Th2, has been reported as another factor contributing to the pathogenesis of GD. Unfortunately, currently available data do not describe the exact mechanism of these subsets’ relation to the GD course and outcome in pediatric patients (12, 13).

Secretion of IL-17 cytokine (mainly an IL-17A) is a crucial feature of the effector Th17 lymphocytes, demonstrating supportive action in the immunity via mobilization of innate and acquired response cells (14). Thus, IL-17-producing cells exert effects that contrast with the regulatory T cells (Tregs) responsible for self-tolerance development and immunosuppression. The disturbed balance of the Th17/Treg interactions has been reported in various autoimmune disorders, including GD, type 1 diabetes, and rheumatoid arthritis (15–19). To date, a few studies have indicated increased levels of Th17 cells in the peripheral blood of GD patients and their participation in orbitopathy (20–22). More recent studies revealed that methimazole-related euthyreosis was accompanied by decreased values of Th17 frequencies in adolescent patients (23).

Although relatively less studied, a subpopulation of effector T cells producing IL-22 (Th22 lymphocytes) has already been shown as a participant in the outcome of autoimmune diseases, also including GD (20, 24–26). To date, conducted studies indicated complicated aspects associated with unambiguous determination of the IL-22 role, as either pro- or anti-inflammatory factor. Nevertheless, IL-22 is predominantly described as one of the main components of the innate immune responses regulating epithelial barrier function. In psoriasis patients, it can stimulate the hyperplasia of epithelium. Moreover, together with IL-17, it can induce proinflammatory processes within bronchial epithelial cells (27). Differentiation and activation of Th22 cells occur within inflamed tissues, with a substantial contribution of IL-6 and TNF-alpha. Besides inflammation-related features, IL-22-producing lymphocytes have demonstrated potential in regenerating and protecting epithelial cells (28). Proper activity of Th22 lymphocytes is essential as their excessive expansion was found to be associated with rheumatoid arthritis, ankylosing spondylitis, and other autoimmune diseases (29, 30).

Thyroid hormones are an essential element of the endocrine system, playing a pivotal role in the body’s development, especially important in children with intensified growth processes. Despite increasing interest in AITDs, there are substantial knowledge gaps regarding the precise influence of the immune cells on inflammatory events. Notably, the previously known cell subpopulations of lymphocytes are constantly being modified. Still, new subpopulations that can significantly impact the pathomechanisms of immune-related diseases were discovered. Our study aimed to evaluate selected effector T lymphocytes, Th1, Th17, and Th22, in newly diagnosed pediatric patients with GD. We additionally determined how therapy with methimazole affects those subpopulations. Most importantly, we established an association between tested lymphocytes and thyroid-related parameters, together with their contribution to the outcome of GD management.

## Materials and methods

### Patient group characteristics

The study was performed in a group of 22 newly diagnosed GD pediatric patients hospitalized in the Clinical Department of Pediatric, Endocrinology, Diabetology with Cardiology Division University Children’s Teaching Hospital in Bialystok and 31 age- and sex-matched healthy control groups without any autoimmune, inflammatory diseases. Patients were treated with methimazole using an adaptive titration regimen, at an initial dose of 0.3–0.6 mg/kg/day in combination with propranolol at 0.5–1.0 mg/kg/day. Peripheral blood (2.7 mL of EDTA-K2 venous blood) was collected at three time points in therapy: before treatment (Time 0), after 3 months (Time 1), and after 12 months (Time 2). Levothyroxine was included in the case of hypothyroidism. Patients were compliant based on the clinical manifestation of GD and results of biochemical parameters such as level of thyrotropin (TSH), free thyroxin (fT4), and free triiodothyronine (fT3). Additionally, the autoantibodies’ anti-receptor for TSH (TRAb) was measured to determine the autoimmune based on hyperthyroidism. The clinical description of analyzed patients is presented in **Supplementary Figure 1**. The local bioethical committee approved the research protocol for the investigation at the Medical University of Bialystok (APK.002.78.2021).

Peripheral blood mononuclear cells (PBMCs) were obtained using density gradient centrifugation with Pancoll (1.077 g/L, Pan Biotech, GmbH, Aidenbach, Germany). PBMC fraction was subsequently washed with PBS without sodium and magnesium (phosphate-buffered saline without Ca2+ and Mg2+; Corning). In the final part, cells were suspended in the cryoprotectant 10% DMSO (Sigma-Aldrich, St. Louis, MO, USA) in FBS (fetal bovine serum, PAN Biotech GmbH, Aidenbach, Germany) and stored in liquid nitrogen until the whole study group’s samples were collected.

### Flow cytometry

After rapid thawing, cells were resuspended in the RPMI 1640 culture medium with 10% FBS and centrifuged per 10 min at 400*g*. Further assessment was followed by counting the cells and confirming their viability. PBMCs were counted on the Bürker chamber using the 0.4% trypan blue solution to determine the amount and viability of cells simultaneously. Only samples containing 95%–100% of viable cells were included in the further assessment. Flow cytometric evaluation of Th1, Th17, and Th22 lymphocytes was performed on 500,000 cells, which were subjected to a 5-hour stimulation with Leukocyte Activation Cocktail containing brefeldin A (BD Pharmingen) at 37°C to enhance the efficiency of intracellular cytokine detection. After incubation, cells were washed with PBS and prepared for fluorometric staining. Firstly, we added a monoclonal antibody to determine CD4 lymphocyte surface markers anti-CD4 FITC (clone RPA-T4). After incubation, unbound antibodies were washed with the PBS solution. Furthermore, to determine intracellular cytokine secretion, we performed permeabilization (Permeabilization Buffer 2, BD Bioscience). The rest of the perm buffer was washed again to allow the staining procedure: anti-IFN-gamma PE-Cy7 (clone B27), anti-IL-17A PE (clone SCPL1362) (BD Bioscience), and anti-IL-22 APC (clone 2G12A41) (BioLegend). The final incubation of cells was followed by twice washing with PBS and fixation of cells using the CellFix Buffer (BD Pharmingen) and stored shortly at +4°C until acquisition. Data were collected using the FACS Calibur flow cytometer (BD Bioscience, Franklin Lakes, NJ, USA) and subsequently analyzed by FlowJo^®^ software (Tree Star Inc., Ashland, OR, USA).

Lymphocytes were determined based on the morphology: size [forward scatter (FSC)] and granularity [side scatter (SSC)], and further CD4+ surface marker presence. Subsequent determination of the mentioned subsets of lymphocytes was based on the positive expression of intracellular markers: IFN-gamma+ for Th1 lymphocytes, IL-17A+ for Th17, and IL-22+ for Th22. Data were presented as a frequency of CD4+ lymphocytes and an absolute number of cells (based on the positive events and normalized in the context of 500,000 cells per test, constant suspension buffer, and acquisition speed, volume, and time). Representative gating strategies with implemented controls are included in **Supplementary Figure 1**.

### Statistical analysis

Statistical assessment of obtained data was performed with GraphPad Prism 9.0 statistical software (GraphPad Prism Inc., San Diego, CA, USA). Firstly, we evaluate the presence of Gaussian distribution of the data. Owing to the lack of it, a nonparametric Mann–Whitney *U* test was applied to compare differences between groups. Moreover, the Wilcoxon test was used for the determination of statistically significant changes in the course of methimazole treatment of pediatric patients with GD. The graphs presented results as median values with interquartile range (IQR). Assessment of the correlation between analyzed parameters was performed with a nonparametric Spearman correlation test. Data were presented as coefficient values (*R*-value), and asterisks indicated significance. The following grouping in the context of data strength evaluation was applied: weak (*r* = 0.20–0.39), moderate (*r* = 0.40–0.59), strong (*r* = 0.60–0.79), and very strong (*r* = 0.80–1.00). Significance level was determined at a *p*-value of 0.05 (*p* = 0.05), and differences were indicated with asterisks or exact *p*-values: *p < 0.05, **p < 0.01, ***p < 0.001, ****p < 0.0001.

## Results

### Initial Th1, Th17, and Th22 lymphocyte levels in Graves’ disease pediatric patients

**Figure 1:**
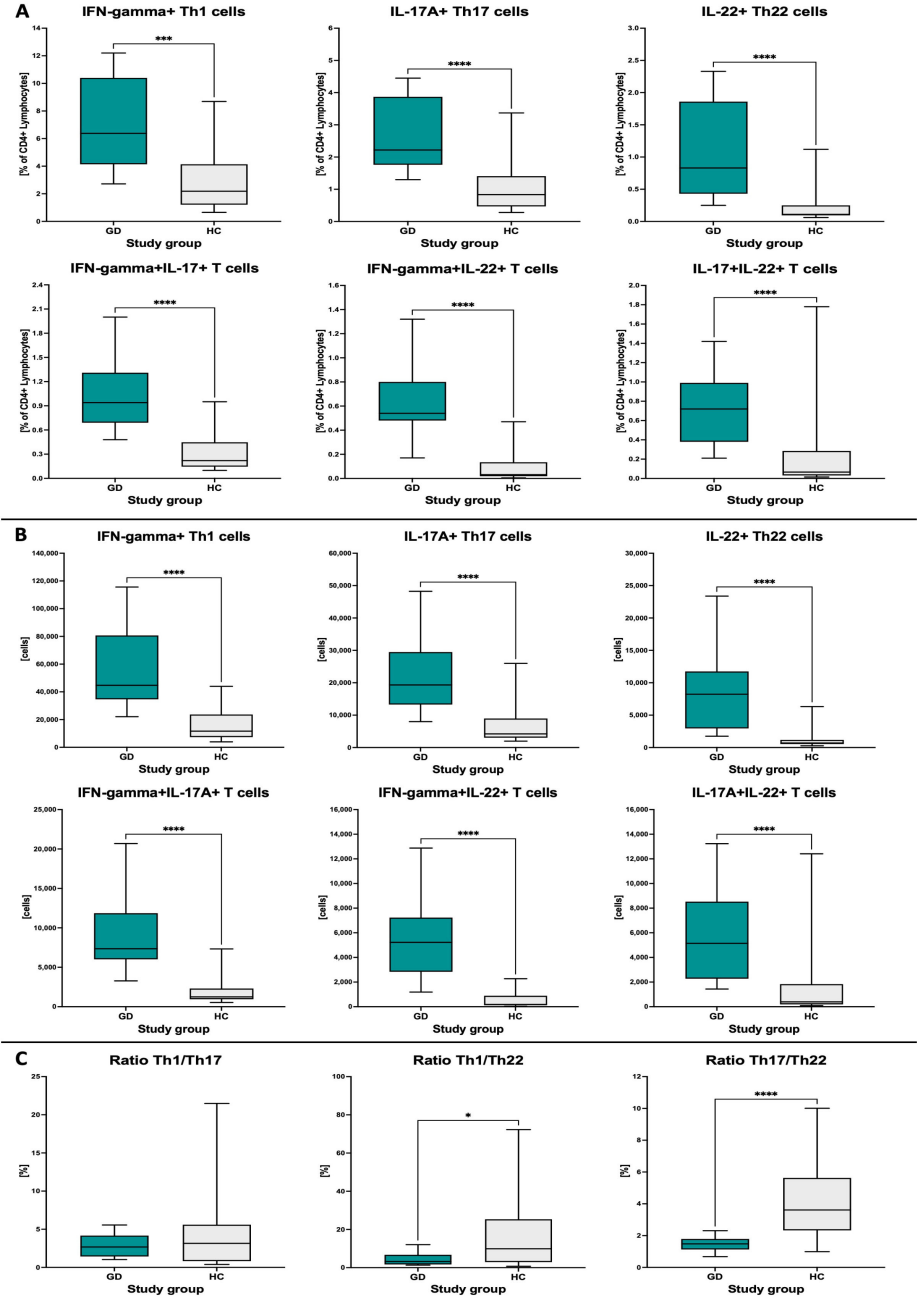
Analysis of differences in Th1, Th17, and Th22 levels between Graves’ disease (GD) patients and the healthy control (HC) group. Data (median values with min to max range) are presented as the frequency of studied populations within CD4+ lymphocytes **(A)**, absolute cell numbers **(B)**, and mutual ratio of cells **(C)**. The significance levels are indicated with asterisks or exact *p*-values: *p < 0.05; ***p < 0.001; ****p < 0.0001.

Firstly, we indicated statistically significant differences between frequencies of Th1, Th17, and Th22 lymphocytes in newly diagnosed GD pediatric patients compared to a healthy control group (HC). In all mentioned subsets of effector T cells, we emphatically determine enhancement values of cells in GD patients. The dominant population was Th1 lymphocytes producing IFN-gamma, constituting our patients’ most significant percentage of CD4+ lymphocytes. Furthermore, we determine the elevated frequencies of lymphocytes simultaneously secreting IFN-gamma and IL-17A (IFN-gamma+IL-17A+), IFN-gamma and IL-22 (IFN-gamma+IL-22+), and IL-17A and IL-22 (IL-17A+IL-22+). Similar to the conventional subsets, we also observed increased frequencies in GD patients (**Figure 1A**). Additionally, changes in the frequency of CD4+ lymphocyte levels were confirmed by assessing the absolute numbers of cells in all the mentioned populations. Here, we also determine that the most significant subpopulations are Th1 lymphocytes. Interestingly, the absolute number of cells secreted by IL-22 was definitely increased in the case of GD patients. In the HC group, negligible levels can indicate the crucial role of Th22 in the onset of GD in pediatric patients (**Figure 1B**). In further assessment, we evaluate the changes in the mutual ratios of the mentioned subsets of effector T cells. Interestingly, we did not observe any statistical changes in the Th1/Th17 ratio. Its level seems identical in both rated groups (GD and HC). Furthermore, considering changes in the Th1/Th22 ratio, we observed that in the HC group, that ratio estimates significant increases compared to GD. Moreover, we also analyzed the Th17/Th22 ratio, which followed a similar tendency but with a substantial enhancement of the importance of observed changes (**Figure 1C**).

### Changes in Th1, Th17, and Th22 lymphocyte subsets during methimazole-based management of Graves’ disease pediatric patients

**Figure 2:**
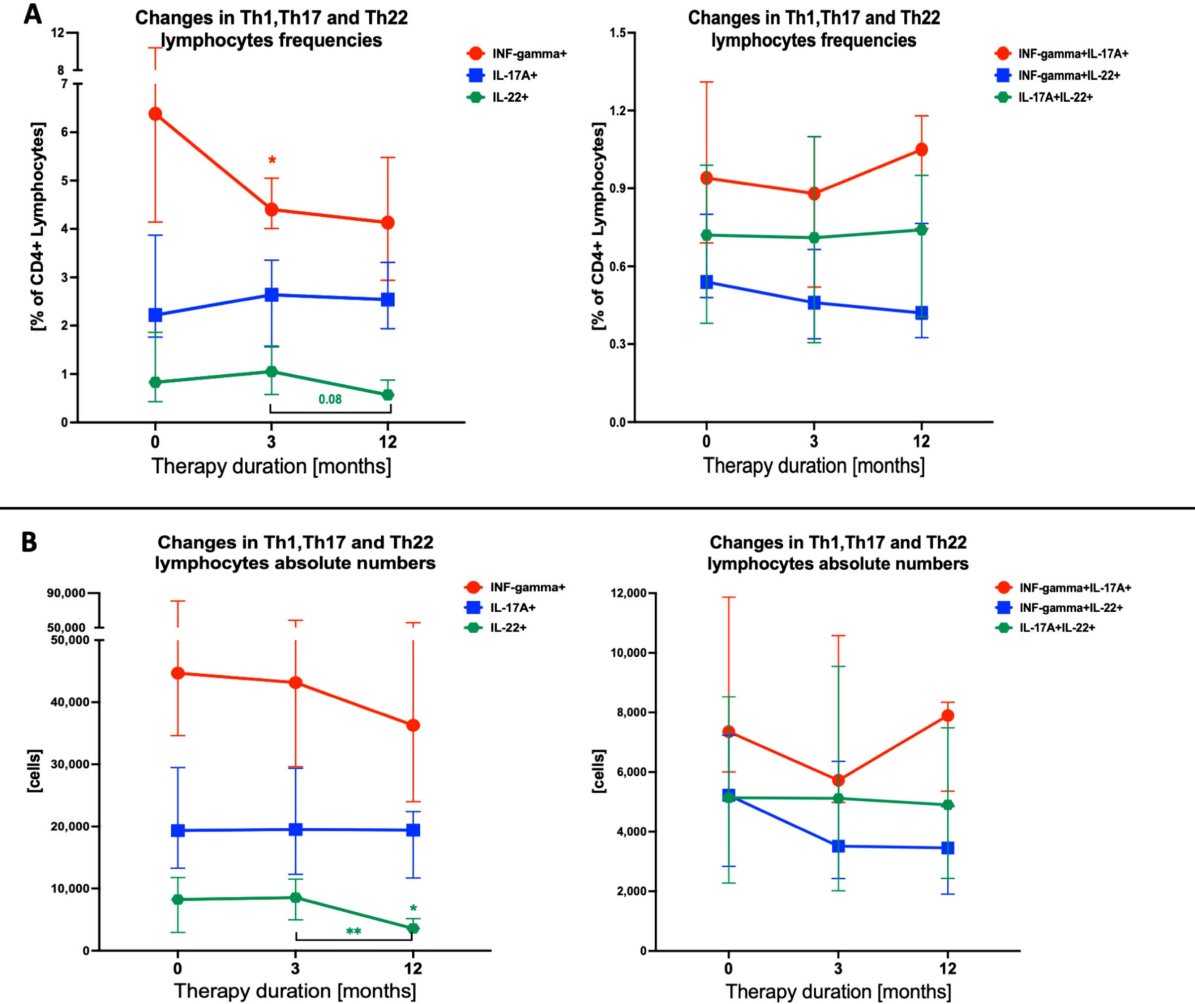
Effects of therapy on Th1, Th17, and Th22 T-cell populations during Graves’ disease management. Data (median with interquartile range) were analyzed as frequency changes of studied populations **(A)** and absolute cell numbers **(B)**. The significance levels indicated with asterisks or exact *p*-values: *p < 0.05; **p < 0.01.

To precisely describe the effect of methimazole on Th1, Th17, and Th22 lymphocyte subpopulations, we assessed their changes throughout therapy, before the implementation of methimazole (Time 0), after 3 months of use (Time 1), and after 12 months of treatment (Time 2). The most responsive population to the applied drug was the secreting IFN-gamma Th1 cells. Since treatment induction, we have observed gradual decreases in the frequency of IFN-gamma+ cells. Moreover, we observed a slight rising trend in Th17 lymphocytes until the 3rd month of treatment, which is normalized at the 12th month of therapy. Importantly, we determined an increasing percentage of IL-22+ lymphocytes (Th22) while simultaneously gradually decreasing its values in long-term treatment (12 months). Further observation was followed by a systematic reduction in frequencies of subsets simultaneously secreting IFN-gamma and IL-22 (IFN-gamma+IL-22+). However, lymphocytes with co-secretion of IL-17A and IL-22 (IL-17A+IL-22+) do not seem to change their frequencies during the methimazole treatment (**Figure 2A**). Considering the changes in frequency levels, we also evaluated the change in the absolute number of cells during the methimazole treatment. Similar to the previous values, we noticed the same trend of changes with its intensification in the case of Th22 cells. We observed a statistically significant decrease in the absolute values of IL-22+ cells during the course of the therapy. Furthermore, the amount of Th17 did not seem to change significantly during treatment, and values of IFN-gamma+ cells presented a similar decreasing tendency, similar to the percentage values. Comparable changes were observed when analyzing absolute numbers of lymphocyte subsets secreting selected cytokines (**Figure 2B**).

### Influences of methimazole treatment for mutual association with Th1, Th17, and Th22 parameters with thyroid function parameters

Assessing the correlations of thyroid parameters with lymphocyte subpopulations Th1, Th17, and Th22 allowed us to observe a positive association between immune parameters and fT3 at admission time. Moreover, we determined a statistically significant positive correlation between the frequency of IL-17A+ and IL-22+ lymphocytes with fT3 values in moderate strength. Simultaneously, TSH and the TRAb autoantibodies’ values present mainly negative correlations with immune-related parameters. It is worth noting that during the determination of the association of the ratio of lymphocyte populations, we observe a total reversal of the observed tendency. The Th1/Th22 ratio positively correlates with TSH in moderate strength. The most significant negative correlations we observed were in the case of fT3, while both Th1/Th17 and Th1/Th22 ratios determined a strong negative association with fT3 values. Furthermore, TSH-R autoantibody (TRAb) values correlate negatively with all immune-related parameters, which is estimated to have the highest strength with the frequency of IFN-gamma+ cells.

**Figure 3:**
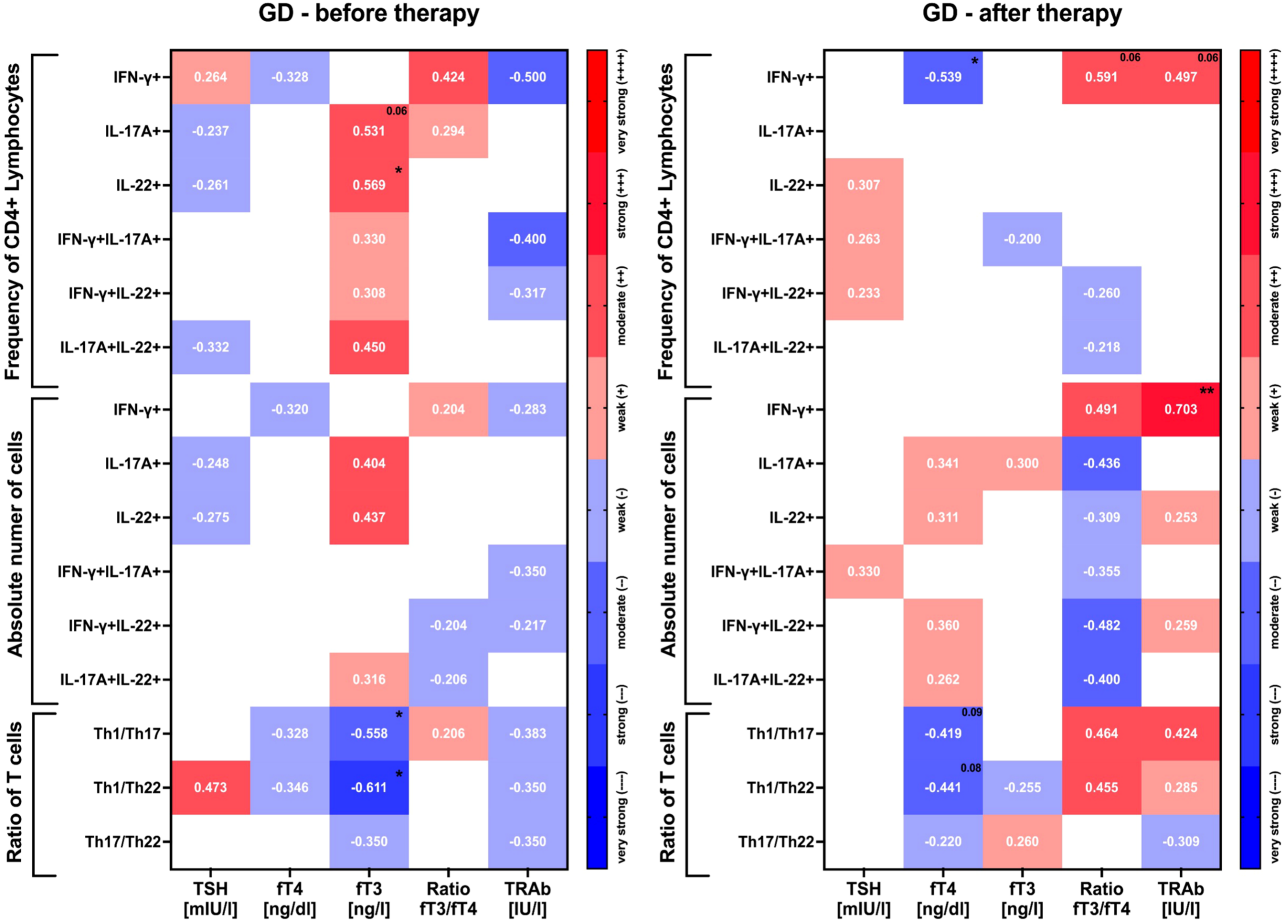
Graphical presentation of correlations between Th1, Th17, and Th22 T cells and thyroid function-related parameters at admission and after therapy. Heatmaps demonstrate *r* values with exact values and color indicating strength subgroup. The following grouping in the context of data strength evaluation was applied: weak (*r* = 0.20–0.39), moderate (*r* = 0.40–0.59), strong (*r* = 0.60–0.79), and very strong (*r* = 0.80–1.00). The significance levels are indicated with asterisks or exact *p*-values: *p < 0.05; **p < 0.01.

The therapy approach intensified the previously established negative correlation between fT4 and IFN-gamma+ cells. Moreover, the frequency of IFN-gamma+ cells also correlated positively with the fT3/fT4 ratio and TRAb values with slight tendency of statistical significance. Interestingly, various negative correlations between immune-related parameters and fT3 values determined before the applied treatment were diminished during therapy. However, moderate-strength negative correlations were noted with immune parameters and the fT3/fT4 ratio. Purely absolute numbers of IFN-gamma+ cells correlate with the fT3/fT4 ratio positively and also with moderate strength. Notably, we indicated an influential association with statistical significance between the absolute numbers of IFN-gamma+ cells and TRAb values. Single positive correlations between the immune-related parameters and the TRAb were determined during treatment. Notably, Th1/Th17 and Th1/Th22 cell ratios presented a negative association with fT4 and correlated positively with the fT3/fT4 ratio and TRAb values (**Figure 3**).

### Monitoring the thyroid-related parameters associated with initial Th1, Th17, and Th22 levels during Graves’ disease therapy

**Figure 4:**
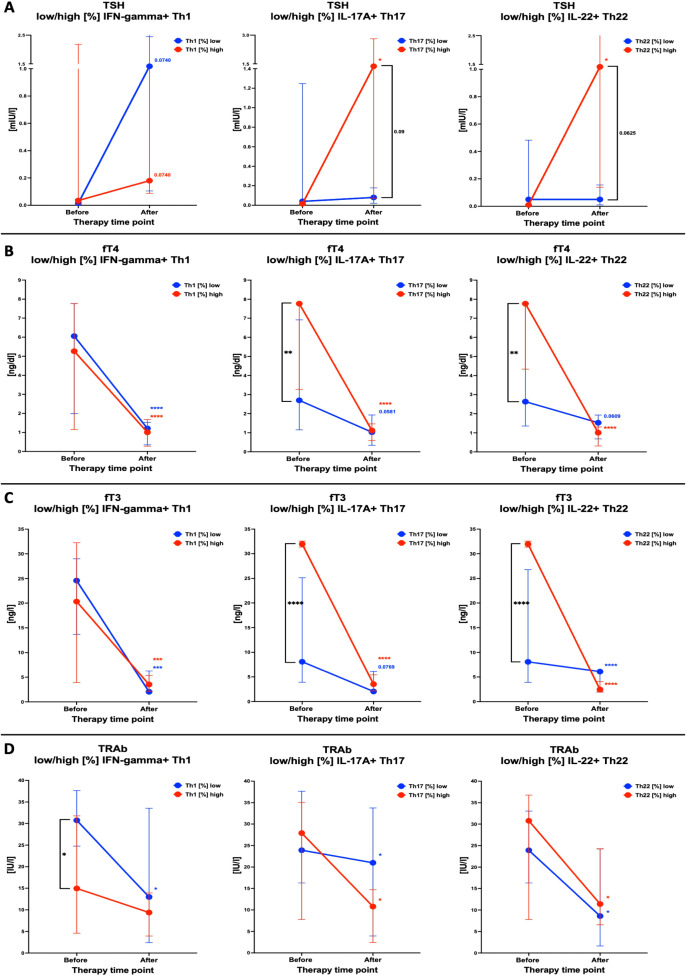
Analysis of changes in thyroid function-related parameters, including stratification of tested lymphocyte populations into patients with a low or high frequency of Th1, Th17, or Th22 T cells. Differences in response were analyzed in the context of TSH **(A)**, fT4 **(B)**, fT3 **(C)**, and TRAb **(D)**. Data are presented as median with interquartile range. The significance levels are indicated with asterisks or exact *p*-values: *p < 0.05; **p < 0.01; ***p < 0.001; ****p < 0.0001.

Considering the probable impact of the initial values of immune cells on the course of treatment, we determined how the initial Th1, Th17, and Th22 frequencies affect the achievement of therapeutic effect when using methimazole. Stratification was based on the median value of Th1, Th17, and Th22 cell frequency within patient groups: low values are below the median, and high values are above the median. Firstly, we obtained normalization of TSH values in patients with an initially lower Th1 lymphocyte frequency, in contrast to subjects with increased Th1 cells, where TSH levels remained unchanged. On the other hand, initially, high percentages of Th17 and Th22 lymphocytes were determined with a statistically significant increase in TSH secretion during therapy. However, observed low frequencies of the mentioned effector T cells resulted in a lack of normalization of the TSH level during treatment. Interestingly, we observed statistically significant differences between low/high frequencies of Th22 based on the TSH values obtained after treatment (**Figure 4A**). Further assessment of the influence of the number of cell subsets depending on the initial low/high level of Th1 lymphocytes shows that the precursory percentage of cells did not significantly affect fT4 and fT3 values. Moreover, during therapy, both subgroups were characterized by a regular decrease in fT4 and fT3, achieving statistically significant decreases and normalization to the reference range level. Nevertheless, statistically significant changes in fT4 and fT3 levels were observed depending on the pre-treatment frequency of Th17 or Th22 lymphocytes in patients. Moreover, both analyzed subsets showed significantly increased fT4 levels, but we observed a substantial reduction in fT4 and fT3 values (back to the reference range) during the therapy approach. In addition, in patients with initially lower lymphocyte values, we observed decreases in fT4 and fT3 during methimazole treatment (**Figures 4B, C**). Finally, we also assessed changes in TRAb levels in the context of initial frequencies of effector T cells. Surprisingly, we observed significant differences in the level of TRAb depending on the increased or decreased percentage of Th1 lymphocytes. Patients with initially lower levels of Th1 lymphocytes were characterized by higher levels of TRAb, which further systematically decreased during therapy, reaching levels corresponding to lymphocytes with higher percentages. Increased frequencies of Th17 and Th22 lymphocyte values at admission time corresponded with higher TRAb levels, which were subsequently accompanied by an effective decrease after methimazole treatment (**Figure 4D**).

### Predictive value of selected lymphocyte subsets in evaluating the effectiveness of therapy in the course of the management of pediatric subjects with Graves’ disease

**Figure 5:**
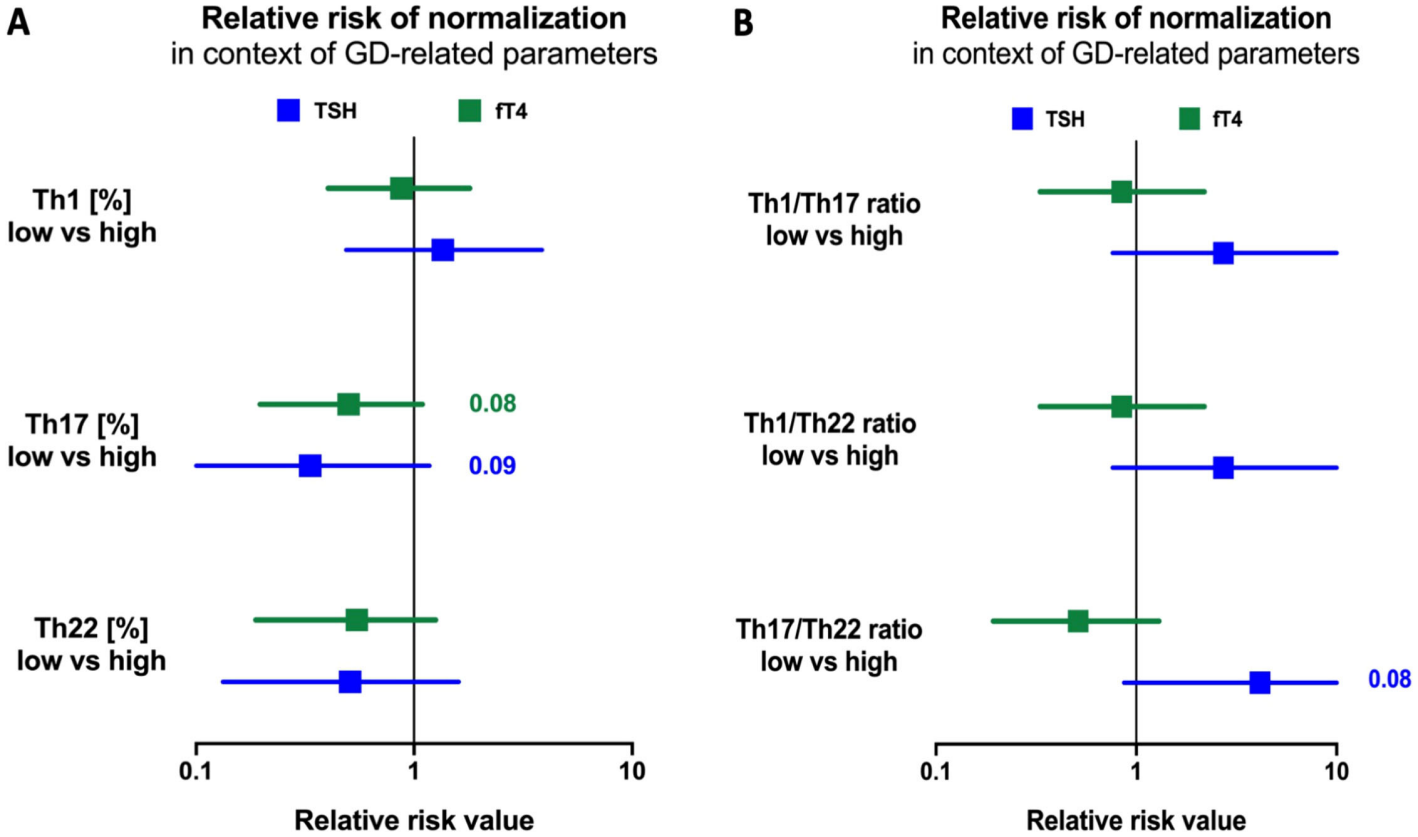
Risk assessment of selected Th1, Th17, and Th22 parameter values for prevalence therapy, frequency of selected lymphocytes **(A)** and their mutual ratios **(B)**. Normalization was achieved based on the selected parameters' normal ranges: TSH (0.32-5.0 mIU/l) and fT4 (0.71-1.55 ng/dl). The significance levels was indicated with exact p values.

Finally, we determined the potential predictive values of selected immune-related parameters in establishing the outcome of GD therapy. For this point, we considered two of the most important clinical parameters in clinical outcomes for the effectiveness of therapy: TSH and fT4. As a normalization range, we established the reference values of selected parameters TSH (0.32–5.0 mIU/L) and fT4 (0.71–1.55 ng/dL). We observed that patients with increased frequencies of Th1 lymphocytes at the initial stage have a higher risk of maintaining the TSH reference value after applied therapy. On the other hand, patients with increased values of Th17 frequencies were determined to have a low risk of failure to obtain an average TSH value (**Figure 5A**). We also assessed if increased/decreased values of effector T cell ratio impact therapy success. We evaluated patients with initially elevated ratios of Th1, Th17, and Th22, followed by a higher risk of maintaining the normal range of TSH after therapy, compared to those with initial lower frequencies (**Figure 5B**).

## Discussion

GD is an organ-specific disease characterized by the failure of self-tolerance mechanisms leading to autoaggressive immune reactions (31). To date, a considerable amount of attention was paid to the B-cell response due to the reported increased production of autoantibodies influencing thyroid tissue. Induced thyroid hormones can stimulate the overexpression of BAFF-activating factors in macrophages, leading to their polarization and activation of B cells. This leads to the lymphocyte differentiation into plasma cells and the development and progression of GD. In accordance, there is a mutual dependence between hormonal and immunological factors contributing to the pathogenesis of autoimmune diseases, including GD (32–34). Noteworthy phenomena in this condition are not limited to the B cells, with T lymphocytes reported as potentially immunomodulating B lymphocytes and, consequently, increasing antibody production (35, 36). Relatively newly discovered subpopulations of effector T lymphocytes, namely, Th17 and Th22, have recently been in the spotlight of the scientific community (27, 37). The manifestation of the disease is undoubtedly associated with infiltration of the thyroid tissue with immunocompetent cells, both B and T cells predominantly (38). Here, we focused on revealing the contribution of Th17 and Th22 subpopulations of T lymphocytes to the disturbance of thyroid function accompanying GD.

At the initial stage of our investigation, we found that GD patients are characterized by a significant increase in values of Th1, Th17, and Th22 effector T cells, compared to HC, both in absolute numbers of cells and their frequency within CD4+ Th lymphocytes. Similarly, Torimoto et al. demonstrated a substantially elevated frequency of Th1 and Th17 lymphocytes in newly diagnosed GD adult patients. Furthermore, a clinical association between the GREAT (Graves Recurrent Events After Therapy) classification and Th1 and Th17 lymphocyte activity levels was presented. It has been shown that a higher frequency of Th17 lymphocytes correlates significantly with the advancement of the pathological process according to the GREAT classification (21). Another group also showed higher Th17 and Th22 cell values in adult subjects with GD, but with no changes in the Th1 subset. The reported changes were additionally supported by concomitant increases in related plasma cytokines, IL-17 and IL-22, respectively, indicating a strong positive correlation between the number of Th17 and Th22, suggesting mutual contribution to the thyroid inflammation (20). This is in agreement with our data showing the most significant changes in the Th17-to-Th22 ratio in pediatric subjects. In addition, we demonstrated dominance of the changes in Th22 over Th17 cells, leading to a decline in Th17/Th22 ratio compared to the HC group. It is worth noting that apart from the blood compartment, increased infiltration of Th17 and Th22 lymphocytes was previously found in thyroid tissue samples from patients with AITDs—GD and Hashimoto thyroiditis (26). The observed phenomenon seems to be age-dependent, as in the study of Song et al., significant elevation of T lymphocyte subsets was exclusively shown only in case of Th22 cells, but the investigation involved only adult GD patients. No changes were found in reference to the IFN-gamma+ (Th1) and IL-17A+ (Th17) T-cell subpopulations. An important addition to our study is the fact that participation of the Th22 cells in thyroid dysfunction was attributed predominantly to the hyperthyroidism related to GD and not hypothyroidism associated with other autoimmune conditions such as Hashimoto (39).

The pharmacological management of hyperthyroidism, involving the use of methimazole (MMI), has been shown as a more convenient and efficient alternative to propylthiouracil, which is used only in several conditions in adult patients due to its hepatotoxicity (40, 41). Despite the confirmed efficacy of MMI in obtaining a euthyroid state, there are ongoing studies on that drug’s actual influence on the immunological phenomenon associated with the autoimmune thyroid dysfunctions. To date, methimazole was found *inter alia* to increase absolute counts of regulatory T cells (Treg) with concomitant reduction of Th17 cells in GD patients (23). Interestingly, methimazole therapy seems to have a minimal effect on levels of Th1, Th17, and Th22 lymphocyte subsets as presented here. Nevertheless, applied therapy reduced the frequency of IFN-gamma+ T lymphocytes in the first 3 months of MMI implementation. Similar effects were shown previously in adults, with a significant decrease in activated Th1 lymphocytes at approximately 6 months of methimazole therapy. Notably, we showed that those effects regarding IFN-gamma-secreting lymphocytes occur during the first 3 months of MMI use, with their normalization at later stages of the therapy. Regarding Th17 cells, this subset of lymphocytes showed no response to the methimazole, as presented in our data (21). Furthermore, IL-22+ T cells showed a tendency for lower absolute numbers after 1 year when compared to the third month of therapy. There are no other data available on Th22 response to MMI in the management of hyperthyroidism. We presume that long-term monitoring of patients might be required to reveal potentially more substantial variations in the tested T-cell populations. This assumption results from the fact that the efficiency of the methimazole therapy (based on the remission rates) gradually increased with years of GD management, from approximately 24% after 1.5–2.5 years to even 75% remission rates after 9 years of the therapy (5). In addition, confirmed participation of Th22 and Th17 cells in the GD outcome might require evaluating its contribution to the other effects associated with MMI application. This includes, among others, the protective influence of methimazole on Treg, which is affected by radioactive iodine (RAI) therapy and causes worsening of the autoimmune reactions (42).

A subset of Th17 cells was demonstrated to exert substantial influence on the severity and duration of the autoimmune thyroid disorders in adult subjects. The involvement of Th17 cells proved to be equally important in the context of orbitopathy occurrence. Th22 lymphocytes seemed to correlate with the level of TRAb antibodies but did not affect the duration of the disease, onset of orbitopathy, or the response to treatment with anti-thyroid drugs (26). Here, the correlation of data obtained at the admission time revealed some crucial associations between tested subsets of lymphocytes and thyroid-related parameters. We showed a close-to-strong positive association between Th17 or Th22 lymphocytes and fT3 level. This is partially in agreement with assessments performed on adult patients. GD subjects showed a weak/moderate positive association of Th17 cells with fT3, as well as fT4, TRAb, and thyroid lobe size (21). Notably, Th1 with Th17 or Th22 ratios correlated negatively with the active form of the thyroid hormone. At the same time, the Th1/Th22 ratio showed a moderate positive correlation with TSH values. Interestingly, no significant associations were demonstrated between tested lymphocyte subsets and TRAb, with moderate negative correlation within subsets expressing IFN-gamma. In contrast, a recent study revealed significant correlations between the percentage of Th17 and Th22 lymphocytes and the level of TRAb, with data corresponding to adult patients in the Chinese population (20). Nevertheless, we showed a negative association of that parameter with the percentage of Th1 lymphocytes, with similar tendency in IFN-gamma+IL-17A+ T cells. The observed trends were reversed in response to treatment implementation. Most importantly, IFN-gamma+ T lymphocytes showed a strong positive association with the blood level of TRAb at the last point of the therapy monitoring. These results complement the above-described reduction in Th1 cells, which seems to be responsible for the concomitant decline in autoantibody values.

We found that initial frequencies of Th1, Th17, and Th22 might play a substantial role in GD patients’ response to the treatment with methimazole. Despite comparable pre-therapy levels of TSH, subjects with higher Th17 or Th22 frequency showed better response with a significant increase of the thyroid parameter. At the same time, high initial levels of those lymphocyte subsets were associated with clearly elevated fT3 and fT4. However, the more intense response of that group to methimazole was later followed by the effective reduction of those hormones. No significant differences in response were found between patients with low and high levels of Th1 cells, with comparably effective reduction of thyroid function-related hormones. Nevertheless, we might presume that higher initial Th1 is instead associated with poor response to therapy, with a complete lack of changes in TSH after the 12th month. In reference to autoantibodies, we found that patients with lower Th1 showed elevated TRAb levels, with significant reduction after therapy to the values comparable to the opposite group. Different Th22 frequencies before methimazole treatment did not influence the efficient reduction of TRAb. Interestingly, GD individuals with high initial Th17 seemed to demonstrate a better response to the therapy with more pronounced decline in those autoantibodies.

In subsequent stages of our investigation, risk assessment analysis showed that patients with higher Th1 frequencies at the beginning of therapy are associated with a higher risk of therapy failure in the context of achieving normal TSH levels on the 12th month of observation. In contrast, higher Th17 or Th22 frequencies were confirmed to contribute beneficially to better outcome prediction based on the normalization of fT4 and TSH. Moreover, our results indicate that patients with initially increased frequency of Th1 lymphocytes are noted with a higher risk of failure of MMI therapy in the context of achieving euthyroidism. However, IFN-gamma+ lymphocytes were the only subsets of effector T cells that showed a statistically significant decline during MMI therapy. Klatka et al. demonstrated an increased value of Th17 lymphocytes resulting from a disturbed balance between Tregs and Th17, which might indicate a short period of disease remission and increased possibilities of occurrences of relapses. Simultaneously, they stated the necessity of prolonging the treatment period to normalize the level of Th17 lymphocytes. In contrast, our study showed that pediatric patients with initially higher Th17 and Th22 values are characterized by a reduced risk of therapy failure, described as normalizing TSH and fT4 levels and, thus, indicating their favorable role as prognostic factors (23).

To the best of our knowledge, we have shown the assessment of Th1/Th17, Th1/Th22, and Th17/Th22 ratios in the onset and management of GD for the first time. Studies from years back showed the crucial role of mutual lymphocyte interactions in the severity of AITD but involved only the Th1/Th2 ratio and its influence on Hashimoto’s disease (38). Differential changes in selected lymphocyte subsets substantially affected the mutual ratio of those cells, namely, Th1/Th17, Th1/Th22, and Th17/Th22 ratios. We observed a reduced ratio of Th1 or Th17 versus Th22 lymphocytes in the group of GD individuals. Significant differences observed exclusively in those parameters, including the Th22 subset, indicate a crucial role of Th22 cells in the inflammatory network involved in the pathogenesis of GD. As mentioned before, noted changes are in favor of higher contribution of Th22 lymphocytes over Th17 in the pathogenesis of GD. Assessment changes in the subsets’ ratio allowed us to evaluate their influence on the clinical aspects of GD. First, we observed that Th1 with Th17 or Th22 ratios correlated negatively with the active form of the thyroid hormone—T3. At the same time, the Th1/Th22 ratio showed moderate positive correlation with TSH values. GD management with methimazole resulted in diminished association reported before therapy implementation. However, we found a tendency for correlation between Th1/Th17 or Th1/Th22 ratio and fT4 levels, presumably related to the frequency of IFN-gamma+ T cells. The previously reported correlation of Th1/Th17 and Th1/Th22 ratios with fT3, after 12 months of therapy, shifted toward their positive association with fT3/fT4 ratio. This aspect seemed to be closely linked to the Th1 values as well considering correlations involving the lymphocyte subset. Additionally, tested lymphocyte ratios were found to be more useful in predicting the normalization of TSH values than fT4. In accordance, lower ratios of Th1/Th17, Th1/Th22, or Th17/Th22 were associated with higher chances of achieving levels of TSH within the normal range after methimazole therapy. Previously described studies have only indicated a positive association between the frequency of Th17 and Th22 lymphocytes and the severity of the disease described using the GREAT or CAS score (21). Our results are the first to note an enhanced chance of achieving a positive therapeutic effect measured via normalization of the thyroid-stimulating hormone according to initially lower values of the Th17/Th22 ratio. The described effect might presumably result from the potentially protective role of Th22 cells. Nevertheless, we are aware of some limitations of our study associated *inter alia* with the lack of related analysis including anti-TSHR autoantibodies. Unfortunately, in subjects with clear signs of remission, based on the clinical features and laboratory basic data (TSH and fT4), assessment of that serological parameter is not commonly implemented at such an early stage of the therapy (12th month).

## Conclusion

GD is an AITD and the most common form of hyperthyroidism in adult and pediatric patients. Despite numerous studies considering the influence of various factors on its pathogenesis, there is still a substantial knowledge gap on the immunological background of this condition. Th1, Th17, and Th22 effector lymphocytes were found to be crucial participants in the disease outcome. Our study indicated significant increases in the frequencies and absolute numbers of selected effector lymphocytes in GD patients. Additionally, we proposed the substantial role of the mutual ratios of Th1/Th17, Th/Th22, and Th17/Th22 cells in this condition. Interestingly, the most susceptible subpopulation of cells that responded to the MMI treatment in the first place was the Th1 subset. However, the obtained results indicate a need for extended observations to possibly report changes also in the context of Th17 or Th22 lymphocytes. Furthermore, low Th17/Th22 ratio values were found to be potential prognostic factors for normalizing TSH levels in response to the methimazole treatment. In addition, long-term monitoring of an immunological remission marker—anti-TSHR—would be essential in future studies. Cumulatively, our data indicate the crucial contribution of Th1, Th17, and Th22 cells in the pathogenesis of GD, together with their great potential in predicting therapy outcomes. Further investigation of larger study groups is of great importance in validating the value of the described immunological parameters in clinical practice. Moreover, follow-up of GD after years of therapy implementation might reveal that Th17 and Th22 lymphocytes influence its outcome through monitoring remission rates.

## Data Availability

The raw data supporting the conclusions of this article will be made available by the corresponding authors, without undue reservation.
