# Supplementary material for: Effect of methimazole treatment on Th1, Th17, and Th22 lymphocytes in pediatric Graves’ disease patients
Source: Front Immunol. 2024 Oct 3;15:1431686. doi: 10.3389/fimmu.2024.1431686 (PMC11494814; doi:10.3389/fimmu.2024.1431686)
Supplement: Supplementary file 1 [file DataSheet1.docx]

Supplementary Material


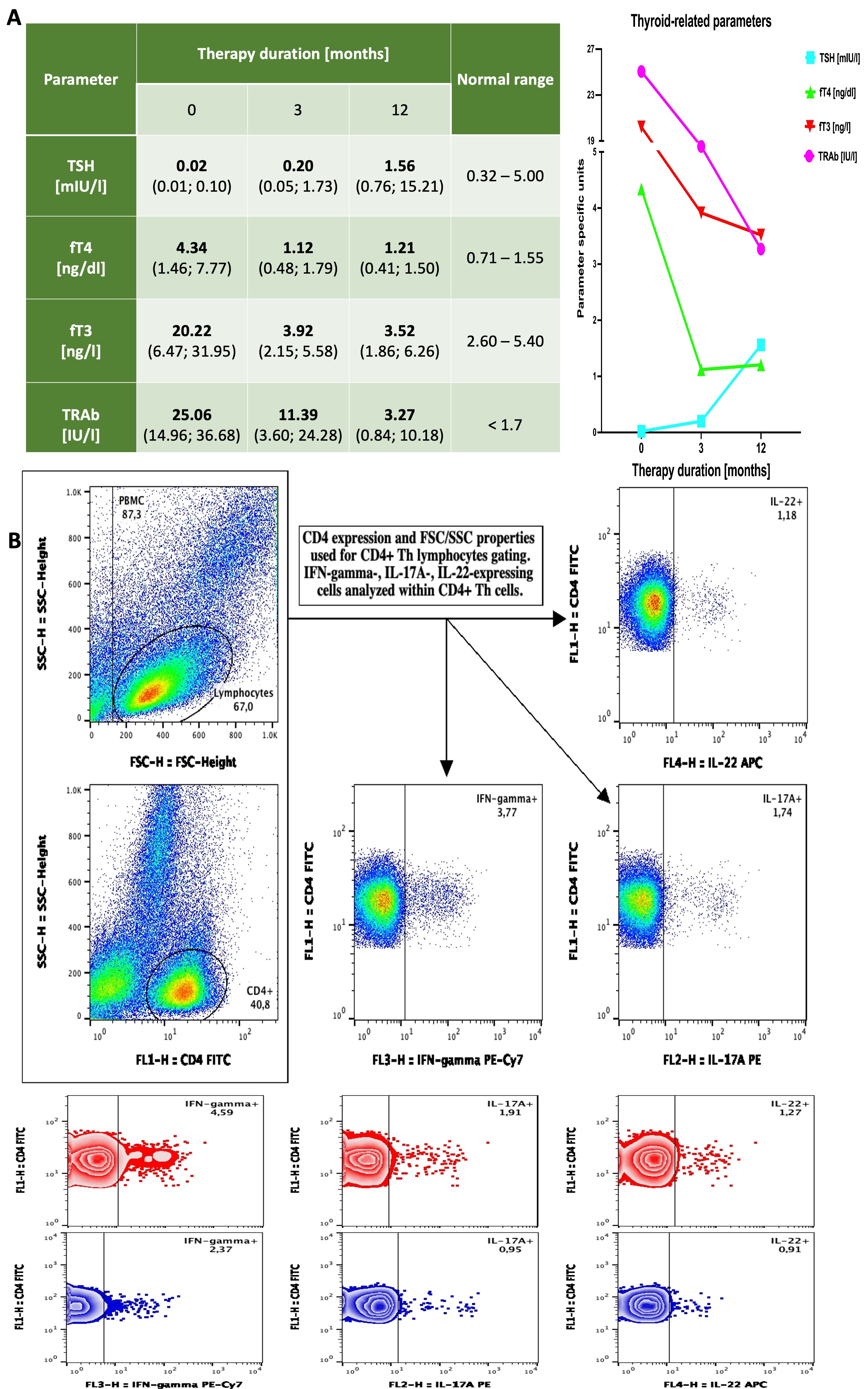


**Supplementary Figure 1.** The clinical characteristic of the studied groups in context of laboratory parameters – Graves’ pediatric patients (GB) during applied MMI therapy. Data presented as median values with 25th and 57th percentile in the brackets **(A).** Gating strategy used in flow cytometric analysis of Th1, Th17 and Th22 cells. Analysis of Th1 cells was based on detection of IFN-gamma production within CD4+ lymphocytes. Th17 cells were distinguished using IL-17A production within CD4+ lymphocytes. Th22 was determine within CD4+ lymphocytes based on the IL-22 secretion. Sample representative plots of tested CD4+ T lymphocyte populations from Graves patients (red) and healthy control group subjects (blue). All the necessary FMO and ISO controls implemented are demonstrated **(B).**
